# The Design and Experiment of a Spring-Coupling Electromagnetic Galloping Energy Harvester

**DOI:** 10.3390/mi14050968

**Published:** 2023-04-28

**Authors:** Lei Xiong, Shiqiao Gao, Lei Jin, Shengkai Guo, Yaoqiang Sun, Feng Liu

**Affiliations:** 1 State Key Laboratory of Explosion Science and Technology, Beijing Institute of Technology, Beijing 100081, China; 13781786703@163.com (L.X.); guoshengkai9@126.com (S.G.); 18712761710@163.com (Y.S.); 2 School of Intelligent Manufacturing, Nanyang Institute of Technology, Nanyang 473000, China; 15838776438@163.com

**Keywords:** spring-coupling, galloping, electromagnetic, energy harvester

## Abstract

In order to improve the output characteristics of the electromagnetic energy harvester in a high-speed flow field, a spring-coupling electromagnetic energy harvester (SEGEH) is proposed, based on the galloping characteristics of a large amplitude. The electromechanical model of the SEGEH was established, the test prototype was made, and the experiments were conducted using a wind tunnel platform. The coupling spring can convert the vibration energy consumed by the vibration stroke of the bluff body without inducing an electromotive force into the elastic energy of the spring. This not only reduces the galloping amplitude, but it also provides elastic force for the return of the bluff body, and it improves the duty cycle of the induced electromotive force and the output power of the energy harvester. The stiffness of the coupling spring and the initial distance between the coupling spring and the bluff body will affect the output characteristics of the SEGEH. At a wind speed of 14 m/s, the output voltage was 103.2 mV and the output power was 0.79 mW. Compared with the energy harvester without a coupling spring (EGEH), the output voltage increases by 29.4 mV, with an increase of 39.8%. The output power was increased by 0.38 mW, with an increase of 92.7%.

## 1. Introduction

Energy harvesting and conversion from wind-induced vibration forms such as galloping [1,2,3], vortex-induced vibration [4,5,6], flutter [7,8,9,10], and multi-vibration coupling [11,12,13] is of great significance in that it can help solve the energy supply of micro-powered electronic devices. Galloping is a self-excited oscillation with a low frequency and large amplitude. The phenomenon of galloping was first proposed by Den Hartog [14], who discovered the galloping phenomenon when analyzing the abnormal vibrations of transmission lines, and he analyzed the causes of galloping. He proposed that when wind speeds exceed 20 miles per hour, transmission lines produce low-frequency, large-amplitude vibrations caused by aerodynamic instability when the slope of the lift curve is greater than that of the drag curve. Parkinson [15,16] studied the determination method of the aerodynamic coefficient in a quasi-steady system, expressed the aerodynamic coefficient as a polynomial form related to the wind speed and the wind attack angle, and conducted experimental verification with square and rectangular cross sections on bluff bodies. Its research results have become the basic theory and method for galloping analyses. Barrero-Gil et al. [17] studied the feasibility of harvesting energy from cross-flow galloping phenomena; for the first time, they found that it has adverse effects on civil engineering and power lines, they established a cross-flow galloping model, and they studied the effects that the cross-section shape, flow velocity, and mechanical characteristics have on energy conversion efficiency. A. Bibo and M. F. Daqaq [18] conducted the dimensionless analysis of the vibration response of the galloping energy harvester and they obtained an approximate analytical solution. The experimental results showed that there is a general relationship between dimensionless output displacement, voltage, power, and flow velocity when under the condition of quasi-steady flow. The general response curve of the galloping energy harvester is established, which is convenient for the optimization analysis of the performance of the galloping energy harvester.

To increase the output of the wind-induced vibration energy harvester, researchers have conducted a large number of experiments and studies on energy harvester structures. Hémon et al. [19] studied the influence of the inclination angle of the prism, relative to the incoming flow direction of the galloping energy harvester in a wind tunnel. Wu et al. [20] studied the influence of the position and length of the piezoelectric sheet and the mass at the end of the cantilever beam on the energy harvester. When the resonant frequency of the energy harvester is close to the vortex shedding frequency, the output power is at its maximum. Zhou et al. [21] designed a wind energy harvesting device consisting of a cantilever beam and a flexible extension structure by imitating the oscillation of leaves in the wind, and they studied the effect of the fan and the T-shaped extension shapes on wind energy harvesting performance. Zhang et al. [22] designed a galloping energy harvester with an asymmetric structure. The cantilever beam was not placed in the middle of the bluff body. Compared with the energy harvester with a symmetrical structure, the starting wind speed was lower during the Monostable operation. Kim et al. [23] proposed a structure for the energy harvester in which two D-shaped bluff bodies are placed opposite each other. The output power of the energy harvester would increase by using the galloping coupling effect of the two D-shaped bluff bodies. Yuan et al. [24] designed a triboelectric nanogenerator driven by wake galloping, which not only has a simple structure, but also has a critical wind speed as low as 1 m/s for collecting wind energy, thus expanding the manner in which energy is harvested via wind-induced vibration. Tan et al. [25] established a hybrid energy harvester model of piezoelectric–electromagnetic synergy, obtained using the approximate analytical solution of the power output of the model, and they took the calculation of the critical wind speed as the criterion for whether the electromagnetic module was put into operation. When the wind speed exceeds the critical wind speed, the electromagnetic module starts to realize the synergistic output of the electromagnetic and piezoelectric modules. The effect is significantly better than the output performance of the single system. Lai et al. [3] placed a rolling rigid mass ball in the bluff body. When the bluff body was galloping, the rigid mass ball rolled left and right and impacted the dielectric elastomer films at both ends, thus resulting in the deformation of the dielectric elastomer and the conversion of vibration energy into electric energy. Wang et al. [26] introduced a nonlinear magnetic force into the structure’s design, which made the structure a tristable working model, the galloping wind speed was reduced to 1 m/s, and its output power was higher under the same wind speed. There are also studies and discussions concerning the energy harvester’s structure, such as the section shape of the bluff body [27,28] and the structure of the cantilever beam [29], and good research results were obtained. Wang et al. [30,31] created a detailed analysis and summary of the relevant research, which is of great value for reference purposes. The modes of wind-induced vibration energy harvesting are piezoelectric [18,32,33], electromagnetic [1], and resonant cavity [34], among which the piezoelectric wind-induced vibration energy harvester has been studied the most. However, the piezoelectric energy harvester has great internal resistance, and it is prone to vibration fatigue. 

This paper proposes a spring-coupling electromagnetic galloping energy harvester (SEGEH). The coupling springs convert the vibration energy of the energy harvester into elastic energy when there is no induced electromotive force in a vibration period; this not only reduces the galloping amplitude, but it also provides an elastic force for the bluff body, and it improves the output power. The proposed output voltage of the SEGEH is first analyzed using the theoretical solution. Subsequently, the output performance of the SEGEH is tested. The experimental results show that the SEGEH can increase the duty cycle of the output voltage, so that the output voltage of the energy harvester always increases with the increase of wind speed, thus allowing the output characteristics to be improved. According to the literature cited by the author, in this study, the SEGEH and the method of increasing the duty cycle to increase the output induced voltage are original.

## 2. Modeling and Analysis

**Figure 1:**
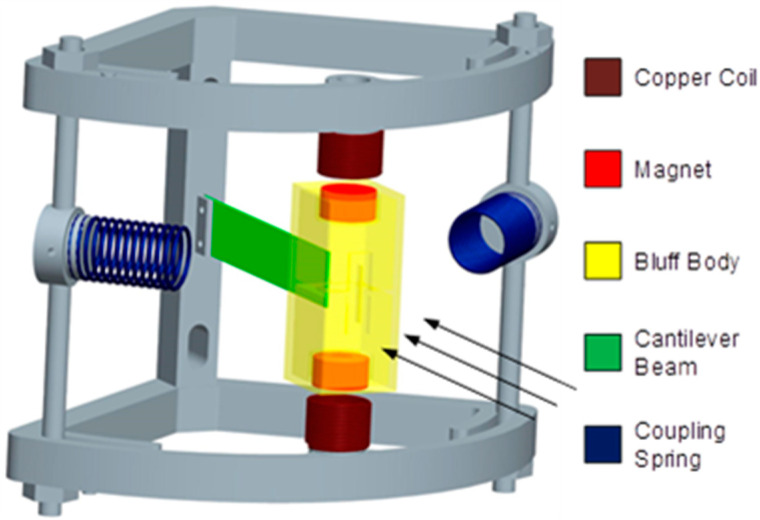
Model diagram of the SEGEH.

The model diagram of the SEGEH proposed in this paper is shown in Figure 1. It consists of an elastic beam, a bluff body, two induction coils, two coupling springs, two permanent magnets, and a bracket. The elastic beam is fixed on the bracket, the bluff body is fixed with the elastic beam, the permanent magnets are embedded into the bluff body at both ends, the coupling springs are symmetrically distributed on the bracket on both sides of the bluff body, and the induction coils are symmetrically arranged on the bracket at both ends of the bluff body. Moreover, the fixed base position is adjustable, with regard to the coupling springs, which is convenient for adjusting the initial distance between the coupling springs and the bluff body during impact, and the impact position can be in the side center of the bluff body.

The motion process of the SEGEH is as follows. The bluff body starts to vibrate under the action of wind, and its amplitude gradually increases with the increase in wind speed until it impacts the coupling spring. Then the bluff body decelerates rapidly. At the same time, under the action of the elastic beam, the bluff body moves in reverse until the coupling spring on the other side impacts it; then, it decelerates rapidly again under the action of the coupling spring and moves in reverse until it hits the coupling spring on the other side, thus forming periodic reciprocal vibrations. During a period of vibration, the vibrations of the bluff body can be divided into two processes: the independent galloping phase before contact occurs between the bluff body and the spring, and the impact coupling vibration phase after contact between the bluff body and the spring.

**Figure 2:**
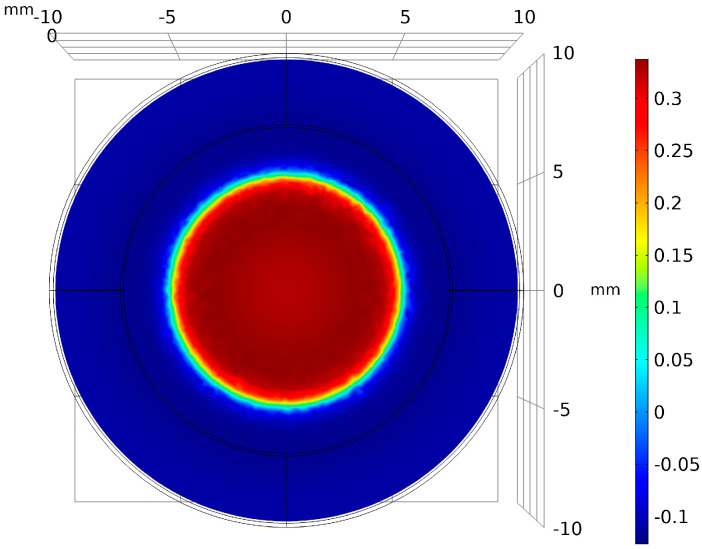
Flux density of the permanent magnet.

The energy harvesting principle of the SEGEH is as follows: galloping is characterized by a large amplitude and low frequency. With the increase in wind speed, the amplitude of the bluff body becomes increasingly larger, which leads to the maximum distance between the permanent magnet and the coil becoming increasingly larger. At the same time, the vibration period becomes increasingly longer, and the vibration frequency becomes increasingly lower. The external magnetic field of the permanent magnet is mainly distributed near the magnet. Figure 2 shows the radial distribution of the magnetic flux density of a cylindrical permanent magnet with a diameter of 10 mm. The magnetic flux density decays quickly after leaving the permanent magnet. Therefore, when the amplitude of the bluff body exceeds the range of the coil, that is, when the permanent magnet and the coil have no intersection area, the flux change rate of the induction coil is approximately zero, and the coil cannot generate an induced electromotive force. The greater the amplitude, the greater the vibration stroke generated by non-induced electromotive forces and the smaller the output duty cycle. Eventually, after reaching a certain wind speed, the induced electromotive force shows a reduction with the increase in wind speed, which greatly limits the output efficiency of the energy harvester.

With the addition of the coupling spring structure, on the one hand, it can limit the amplitude of the bluff body, control the maximum distance between the induction coil and the permanent magnet, reduce the vibration stroke and vibration time generated by the non-induced electromotive force, and improve the output duty cycle of the induced electromotive force. On the other hand, the coupling spring can convert the vibration energy consumed by the vibration stroke, without the induced electromotive force output of the bluff body, into the elastic energy of the spring, which provides an elastic force for the return of the bluff body, increases the vibration speed, and improves the induced electromotive force and vibration frequency. If the energy loss of the coupling spring itself is ignored, the vibration energy of the bluff body will remain unchanged in each vibration period, and the energy harvesting efficiency will be improved. Therefore, the coupling spring can limit the amplitude of the energy harvester, shorten the vibration period, and improve the vibration frequency and induced electromotive force.

**Figure 3:**
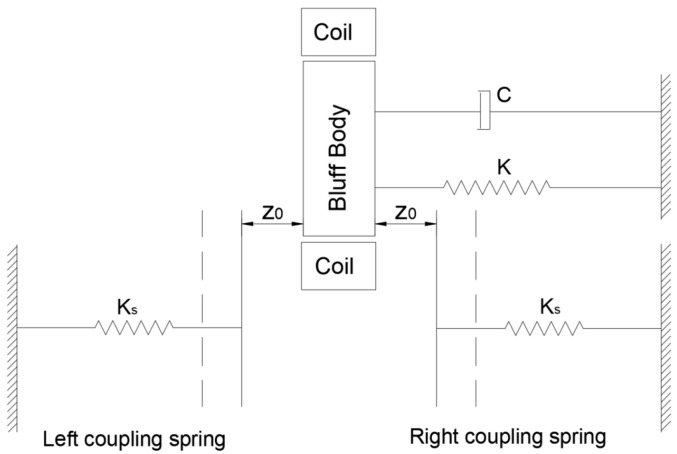
Simplified model of the SEGEH.

**Figure 4:**
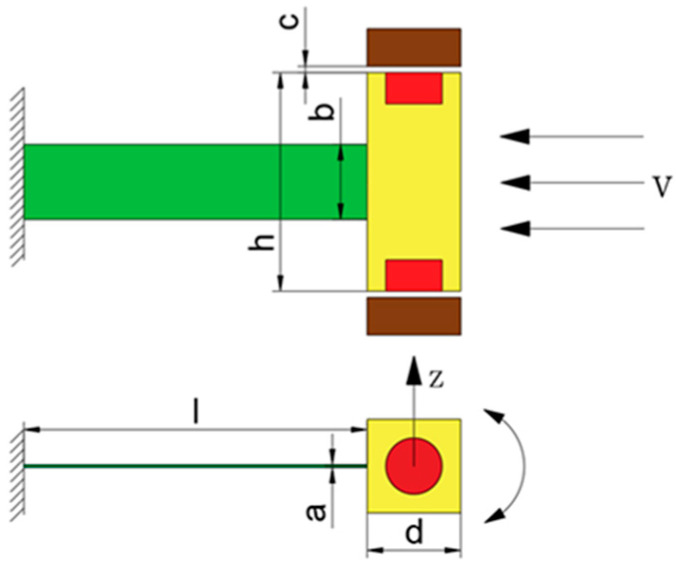
Vibration structure model of SEGEH.

**Figure 5:**
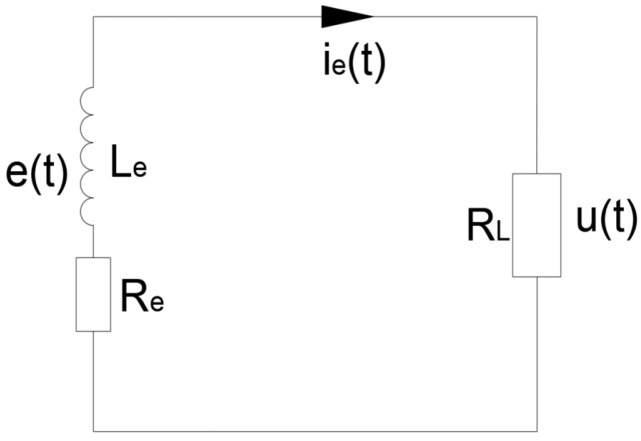
Schematic diagram of induction coil circuit.

Under the action of the wind, SEGEH forms a periodic vibration system composed of a cantilever beam and coupling spring. The system can be equivalent to a spring- cantilever beam-mass system, and the simplified model is shown in Figure 3. Z_0_ represents the initial distance between the coupling spring and the bluff body; m, C, and K are the equivalent mass, damping coefficient, and elastic coefficient of the cantilever structure, respectively; the coupling springs on both sides are an independent single-degree-of-freedom spring, and the elastic coefficient is K_s_. When the cantilever beam is in the initial equilibrium state, the central axis of the bluff body is taken as the origin of the Z direction of the galloping motion, as shown in Figure 4. Figure 5 is the equivalent circuit diagram of the SEGEH. R_L_ is the load resistance; L_e_ and R_e_ are the resistance and inductance of the induction coil, respectively; *e(t)* is the induced electromotive force generated by the coil. *u(t)* is the load voltage; *i_e_(t)* is the loop current.

The governing equations of the SEGEH’s electromechanical model can be expressed as per Equation (1):

(1) $$\left\{\begin{array}{l} m\ddot{z}(t)+C\dot{z}(t)+Kz(t)=F_{g}-F_{e}(t)-F_{s}(t)\\ L_{e}\frac{di_{e}(t)}{dt}+(R_{e}+R_{L})i_{e}(t)+e(t)=0 \end{array}\right.$$

where *F_e_(t)* is the electromagnetic damping force produced by the induced current on the bluff body; *F_g_* is the aerodynamic force on the bluff body; *F_s_(t)* is the elastic force generated by the coupled spring on the bluff body; $C=2\xi \sqrt{\mathrm{mK}}$, ξ and ω are the damping ratio and natural frequency of the cantilever beam structure, respectively. *e(t)* is the induced electromotive force, which is related to the vibration displacement and vibration speed of the bluff body.

The aerodynamic force on the bluff body under the action of wind with speed *V* can be expressed as [15,16]:

(2) $$F_{g}=QV^{2}[A_{1}(\dot{z}/V)+A_{3}{\left(\dot{z}/V\right)}^{3}]$$

where ρ is the air density; Q=(ρdh)/2, d and h are the length of the windward surface of the bluff body; A1 = 2.69 and A3 = −168.4 are the empirical coefficients of galloping [35]. 

The electromagnetic damping force on the bluff body is:

(3) $$\begin{array}{cc} F_{e}(t)=\frac{\partial W}{\partial z}=\frac{\partial \Phi }{\partial z}i_{e}(t)=\mathrm{nB}\frac{\partial S}{\partial z}i_{e}(t)=g_{e}i_{e}(t) & \left|z(t)\right|\leq 2r \end{array}$$

where *W* is the virtual work conducted by the current-carrying coil in the magnetic field. The radius of the coil and the permanent magnet is equal, and thus, both are r. As shown in Figure 2, the magnetic field of the permanent magnet is only distributed around the circumference. When the permanent magnet does not intersect with the coil, its flux change rate is approximately 0. Therefore, only when *|z(t)|* ≤ 2r does the coil induce an electromotive force, and the bluff body is affected by the electromagnetic damping force *F_e_(t)*.

When *|z(t)|* > Z_0_, the bluff body impacts the coupled spring, the bluff body is subjected to the elastic force of the coupled spring *F_s_(t)*, and it quickly slows down and returns. The elastic force on the bluff body is:

(4) $$\begin{array}{cc} F_{s}(t)=K_{s}(z(t)-Z_{0}) & \left|z(t)\right|>Z_{0} \end{array}$$

The induced electromotive force generated by the coil is:

(5) $$\begin{array}{cc} e(t)=n\frac{d\Phi }{dt}\approx -\mathrm{nB}\frac{\partial S}{\partial t}=-\mathrm{nB}\frac{\partial S}{\partial z}\dot{z}(t)=-g_{e}\dot{z}(t) & \left|z(t)\right|\leq 2r \end{array}$$

where n is the number of the coil turns, and B is the average flux density. $g_{e}=\mathrm{nB}\frac{\partial S}{\partial z}$, is the electromechanical coupling coefficient. The vibration frequency of the energy harvester is less than 10 Hz, so the inductive effect can be ignored. Equation (1) can be simplified as follows:

(6) $$(R_{e}+R_{L})i_{e}(t)+e(t)=0$$

Let us substitute Equations (2)–(6) into Equation (1). In accordance with the force change of the bluff body, the vibration process is thus divided into three sections. The governing Equation (1) can be expressed as:

(7) $$\begin{array}{cc} \left\{\begin{array}{l} \ddot{z}(t)+\frac{1}{m}(C-A_{1}QV)\dot{z}(t)+\frac{K}{m}z(t)+g_{e}i_{e}(t)\\ -\frac{A_{3}Q}{mV}(\dot{z}(t){)}^{3}=0\\ (R_{e}+R_{L})i_{e}(t)-g_{e}\dot{z}(t)=0 \end{array}\right. & \left|z(t)\right|\leq 2r \end{array}$$

(8) $$\left\{\begin{array}{ll} \ddot{z}(t)+\frac{C-A_{1}QV}{m}\dot{z}(t)+\frac{K}{m}z(t)-\frac{A_{3}Q}{mV}{\left(\dot{z}(t)\right)}^{3}=0 & 2r<\left|z(t)\right|\leq Z_{0}\\ \begin{array}{l} \ddot{z}(t)+\frac{C-A_{1}QV}{m}\dot{z}(t)+\frac{K+K_{s}}{m}z(t)\\ -\frac{K_{s}}{m}Z_{0}-\frac{A_{3}Q}{mV}{\left(\dot{z}(t)\right)}^{3}=0 \end{array} & \left|z(t)\right|>Z_{0} \end{array}\right.$$

Equation (7) can be simplified as:

(9) $$\begin{array}{cc} \begin{array}{l} \ddot{z}(t)+\frac{1}{m}(C+\frac{g_{e}{}^{2}}{R_{e}+R_{L}}-A_{1}QV)\dot{z}(t)+\frac{K}{m}z(t)\\ -\frac{A_{3}Q}{mV}{\left(\dot{z}(t)\right)}^{3}=0 \end{array} & \left|z(t)\right|\leq 2r \end{array}$$

When *|z(t)|* ≤ 2r, the coil has induced an electromotive force, and the bluff body is affected by the aerodynamic force *F_g_* and electromagnetic damping force *F_e_(t)*. When 2r < *|z(t)|* ≤ Z_0_, the induced electromotive force of the coil is approximately 0, and there is no contact between the bluff body and the coupled spring. At this time, it is only affected by the aerodynamic force *F_g_*. When *|z(t)|* > Z_0_, the bluff body impacts the coupled spring, and the bluff body is subjected to the aerodynamic force *F_g_* and the elastic force of the coupled spring *F_s_(t)* at the same time. It is evident that the larger the amplitude of the energy harvester, the greater the displacement without an induced electromotive force. Therefore, reducing the amplitude can improve the efficiency of the energy harvester. The equations were solved by the fourth-order Runge–Kutta method of numerical calculation software.

**Figure 6:**
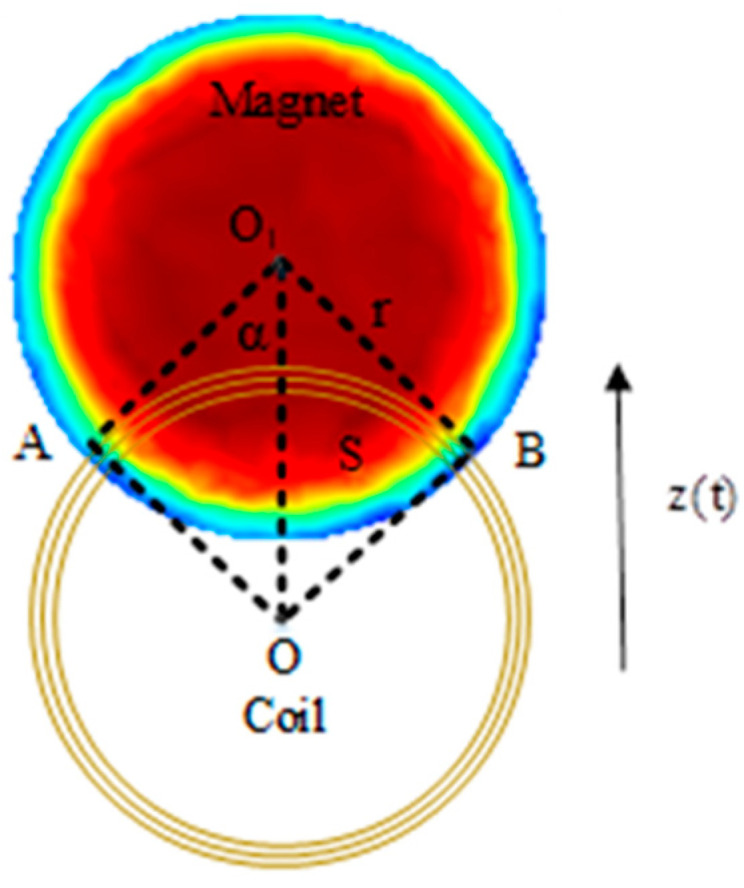
Intersection area S between the induction coil and the permanent magnet. A and B are the projected intersection points of the coil and the magnet. O and O_1_ are the centers of the coil and the magnet, respectively.

Figure 6 is a schematic diagram of the intersection area S between the induction coil and the permanent magnet. Where *z(t)* is the displacement of the bluff body, and is approximately equidistant OO_1_ from the coil and the permanent magnet. α and S are expressed by Equations (10) and (11), respectively.

(10) $$\alpha =\arccos\left[z(t)/2r\right]$$

(11) $$s=2r^{2}\alpha -rz(t)\sin\alpha$$

When the energy harvester is working, the permanent magnet vibrates periodically, relative to the coil, and the induced electromotive force generated by the coil is:

(12) $$g_{e}=\mathrm{nB}\frac{\partial S}{\partial z}=\mathrm{nB}\frac{\partial \left[2r^{2}\alpha -r\sin\alpha z(t)\right]}{\partial z}=-\mathrm{nB}\sqrt{4r^{2}-z^{2}(t)}$$

The load voltage *u(t)* is:

(13) $$\begin{array}{cc} u(t)=R_{L}i_{e}(t) & \left|z(t)\right|\leq 2r \end{array}$$

## 3. Experiment and Discussion

In order to explore the influence of the coupling springs on the output performance of the prototype, this study first conducted experiments and analyzed the designed EGEH; then, we selected nine springs with different parameters for coupling impact experiments. In accordance with the experimental results, the 0.6 × 12 × 25 spring was selected as the coupling spring of this experiment. Finally, experiments and an analysis of the output characteristics of the SEGEH were conducted in detail.

### 3.1. Experimental Setup of the SEGEH

**Figure 7:**
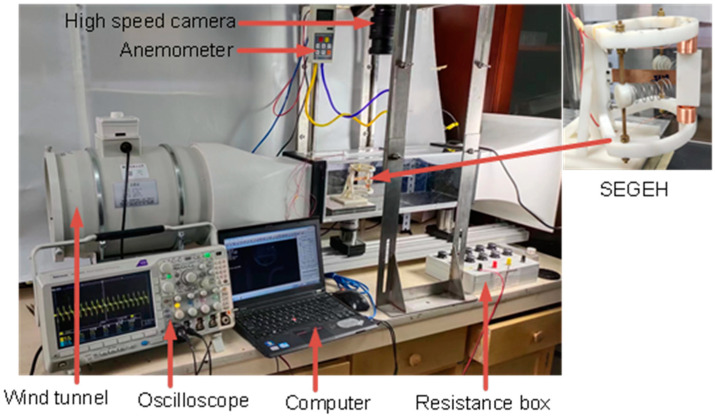
Experimental setup of the SEGEH.

Table 1 shows the material and geometric parameters of the experimental model. Figure 7 shows the physical diagram of the experimental setup. The experiments were conducted using the laboratory wind tunnel experimental platform (Model KT-03, produced by Wangai Teaching Equipment Co., Ltd, Shanghai China), which is capable of continuously regulating speed in a wind speed range of 2~14.5 m/s. Real-time wind speed monitoring was carried out by an anemometer (Model DP3000, produced by Yiou Instrument Equipment Co., Ltd, Shanghai China). The output voltage was measured by the oscilloscope (Tektronix: MDO3024, produced by Tektronix Inc, USA). The material of the elastic beam was beryllium bronze; the material of the bluff body was resin, with a hollow design and 3D printing processing; the material of the permanent magnet was NdFeB; and the material of the coupling spring was 304 stainless steel.

### 3.2. Output Response of the EGEH

**Figure 8:**
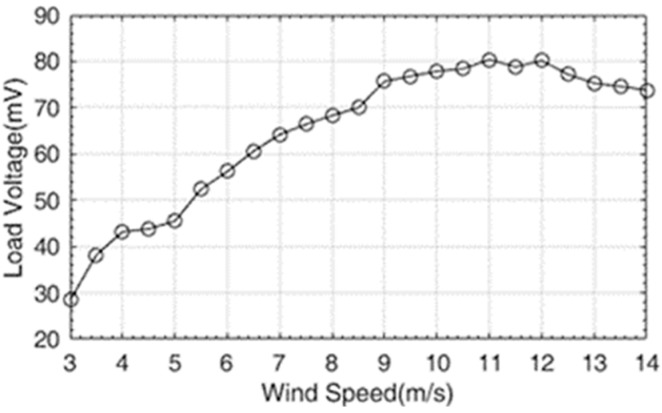
The relationship between the load voltage of the EGEH and wind speed.

**Figure 9:**
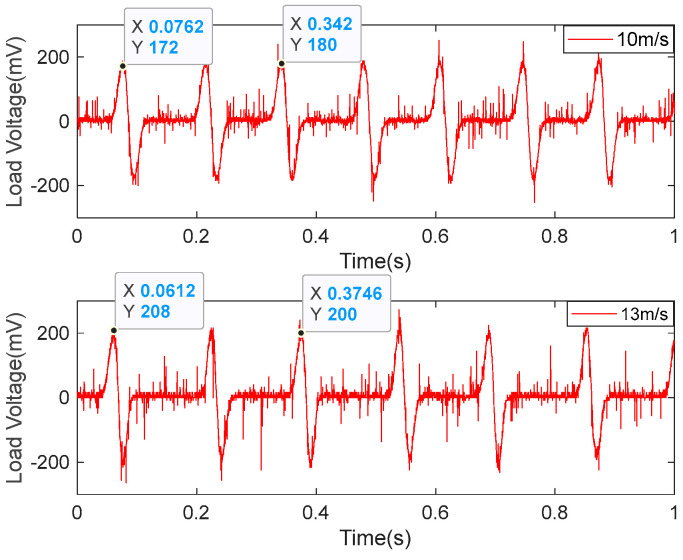
Load voltage waveforms of the EGEH at 10 m/s and 13 m/s.

Figure 8 shows the relationship between the output voltage of the EGEH and wind speed. When the wind speed was less than 9 m/s, the output voltage increased rapidly with the increase in wind speed; when the wind speed was 9~12 m/s, the output voltage changed slowly with the increase in wind speed; and when the wind speed was greater than 12 m/s, the output voltage showed a reverse trend, and it decreased with the increase in wind speed. The output voltage of the energy harvester first increased and then decreased with the increase in wind speed. If the wind speed exceeded 9 m/s, the output characteristics of the energy harvester would have wasted a large amount of wind energy, which would have limited the output efficiency of the energy harvester. In order to improve the output performance of the energy harvester, the output characteristics of the wind speed at 10 m/s and 13 m/s were analyzed. Figure 9 shows the output voltage waveforms measured by the EGEH at 10 m/s and 13 m/s wind speed, respectively. At this point, the RMS voltages were 77.9 mV and 75.3 mV, the peak voltages were 180 mV and 200 mV, and the periods were 0.26 s and 0.31 s, respectively. The energy harvested in each period was 5.97 × 10^−5^ J and 6.66 × 10^−5^ J. The harvested energies within 1 s were 2.3 × 10^−4^ J and 2.15 × 10^−4^ J, respectively. At a wind speed of 13 m/s, more energy was harvested in each period and the peak voltage was greater; however, the RMS voltage decreased. This is because the duty cycle of the output voltage decreased, from 42% of 10 m/s to 33% of 13 m/s. Although the vibration period increased by 0.05 s, the output time was reduced from 0.108 S to 0.102 S, and thus, the vibration period had no output. As a result, less energy is captured in a second. The fundamental reason for the decrease in the duty cycle is that the amplitude of the bluff body was too large. The excessive amplitude caused the distance between the induction coil and the magnet of the energy harvester to exceed the minimum distance that the coil could generate, with regard to the induced electromotive force. Therefore, the excessive amplitude is the main reason that the output performance of the energy harvester decreased under medium and high wind speed. 

### 3.3. Influence Analysis of the Coupling Springs

**Figure 10:**
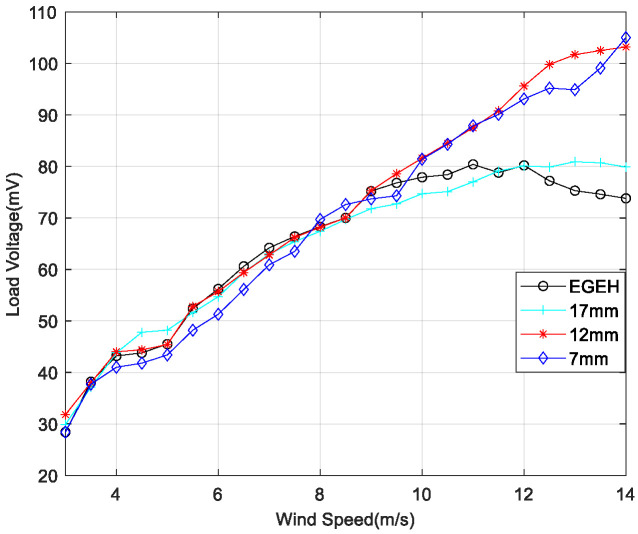
Load voltage with different initial distances.

**Figure 11:**
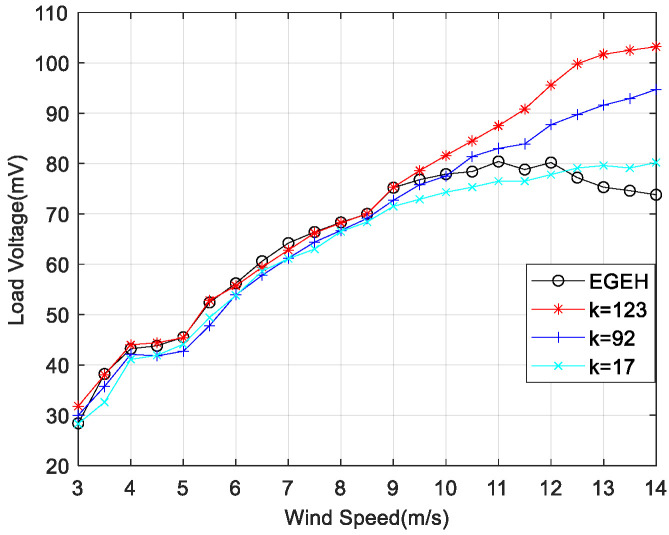
Load voltage with different stiffness.

In order to reduce the amplitude of the energy harvester and improve its output performance, a spring-coupling electromagnetic energy harvester (SEGEH) was proposed. In order to evaluate the influence of the coupling spring on the output performance of the energy harvester, nine different springs were selected for coupling impact experiments. The fixed positions of the springs remained the same—all were at a 55 degree arc from the center. All nine SEGEHs improved the output performance of the energy harvester, with a maximum load voltage increase of 42% and a minimum load voltage increase of 9%, at a maximum experiment wind speed of 14 m/s. The main factors influencing the improvement are as follows. First, the initial distance between the coupling spring and the bluff body has a great influence on the coupling collision and the improvement of the output critical wind speed. Figure 10 shows the output comparison of 0.6 mm wire diameter springs with the same stiffness, and initial distances of 17 mm, 12 mm, and 7 mm, respectively. The initial distance corresponds with the spring lengths of 20 mm, 25 mm, and 30 mm, respectively. The critical wind speeds for the coupling impact were 10 m/s, 7.5 m/s, and 6 m/s, respectively, and the minimum critical wind speeds for improved output were 11.5 m/s, 9 m/s, and 8 m/s, respectively. The output for the initial distances of 12 mm and 7 mm is significantly better than that at 17 mm. However, it should be noted that if the initial distance is too small, its disturbance to the airflow will reduce the output performance before the impact. Second, regarding the stiffness of the coupling spring, the higher the stiffness of the spring, the better its coupling performance. Figure 11 shows the output comparison of three kinds of springs with different stiffnesses. When the initial distance was 12 mm, the output voltage of the 0.6 × 25 mm spring with a stiffness coefficient of k = 123 was significantly better than other springs. The maximum increase amplitude of the output voltage after coupling was 40%. The coupling wind speed rangd from 9 m/s to 14 m/s. After a comprehensive comparison, the 0.6 × 12 × 25 mm spring was selected as the coupling spring for the SEGEH in this study, which can improve the output performance after a coupling collision with almost no disturbance before coupling. The SEGEH in the following refers to the SEGEH used this spring.

### 3.4. Output Response of the SEGEH

**Figure 12:**
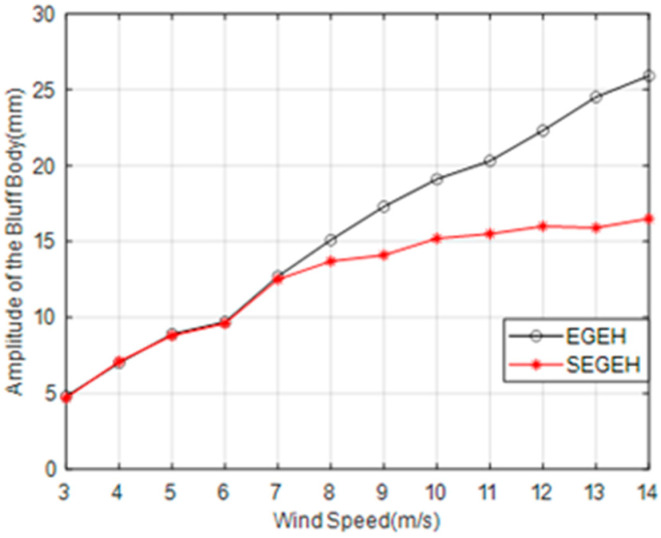
Amplitude comparison of the SEGEH and the EGEH.

Figure 12 shows the amplitude variation curve of the SEGEH. The amplitude of SEGEH increases slowly after coupling impact. When the wind speed increased from 8 m/s to 14 m/s, the SEGEH amplitude increased from 13.7 mm to 16.5 mm—an increase of 2.8 mm. The amplitude of the EGEH increased by 10.8 mm from 15.1 mm to 25.9 mm. The amplitude was reduced by up to 9.4 mm—a reduction of 36%. Moreover, we also took the output characteristics at 10 m/s and 13 m/s wind speed in order to study.

**Figure 13:**
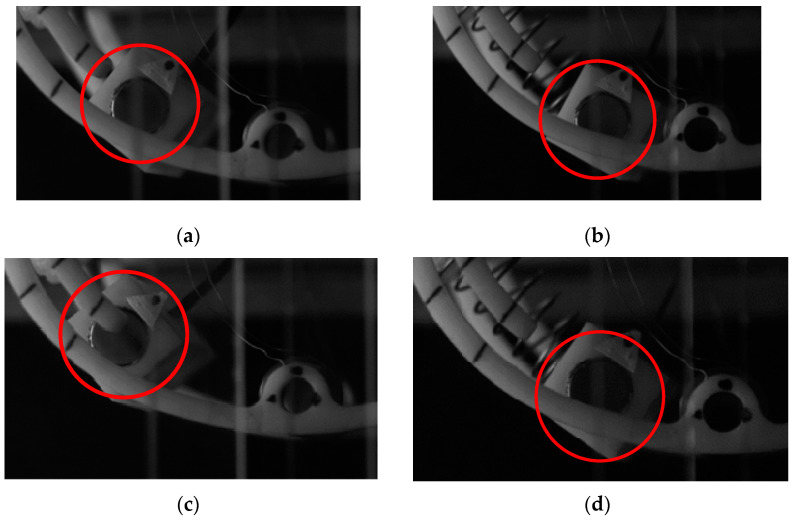
Amplitude variation of the SEGEH captured by a high-speed camera; (**a**) amplitude of the EGEH at 10 m/s; (**b**) amplitude of the SEGEH at 10 m/s; (**c**) amplitude of the EGEH at 13 m/s; (**d**) amplitude of the SEGEH at 13 m/s.

**Figure 14:**
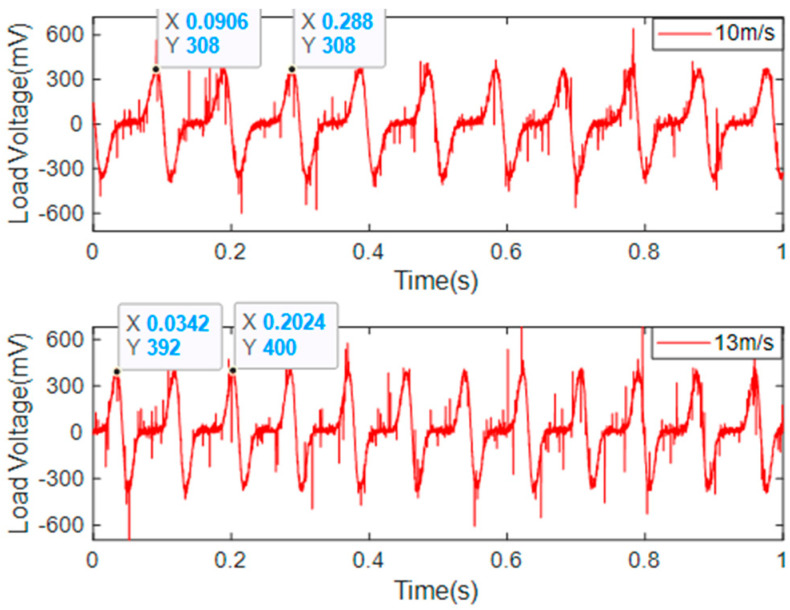
Load voltage waveforms of the SEGEH at 10 m/s and 13 m/s.

Figure 13 shows the amplitude of the SEGEH and the EGEH at 10 m/s and 13 m, respectively/s, as taken by high-speed camera. The amplitudes of the SEGEH were 15.2 mm and 15.9 mm, respectively, demonstrating an increase of 0.7 mm. The EGEH amplitudes were 19.1 mm and 24.5 mm, respectively, demonstrating an increase of 5.4 mm. SEGEH's amplitude increment is 4.7mm less than EGEH's. That means, after adding the coupling springs, the distance that the energy harvester can move is 18.8 mm less during a vibration period. According to Equation (5), the induced electromotive force is related to its amplitude. When the amplitude of the bluff body exceeded 10 mm, the flux change rate through the coil was approximately 0, and the induced electromotive force was no longer generated. At a wind speed of 10 m/s, the amplitude reached 19.1 mm. Therefore, the 18.8 mm displacement of 13 m/s did not generate an induced electromotive force. This is also the reason why the output duty cycle of the EGEH gradually decreased. However, the amplitude of the SEGEH changed little after 9 m/s, which reduced the vibration displacement without an induced electromotive force and improved the output duty cycle. Figure 14 shows the output voltage waveforms of the SEGEH at wind speeds of 10 m/s and 13 m/s, respectively. At this point, the energy harvested by the energy harvester in each period was 5.04 × 10^−5^ J and 6.27 × 10^−5^ J, respectively. Compared with the case without springs, although the harvested energy in a period does not increase, the period is shorter, so the output voltage increased by 3.7 mV and 26.4 mV, thus resulting in 81.6 mV and 101.7 mV, respectively, with an increased range of 4.7% and 35.1%, respectively. The main reason for this increase is that the output duty cycle is improved, which is 65.6% and 66.2%, respectively, and the output frequency increased to 5 Hz and 6.1 Hz. Within 1 s, the harvested energy increased to 2.52 × 10^−4^ J and 3.92 × 10^−4^ J, respectively. Therefore, the design scheme of the SEGEH is effective.

**Figure 15:**
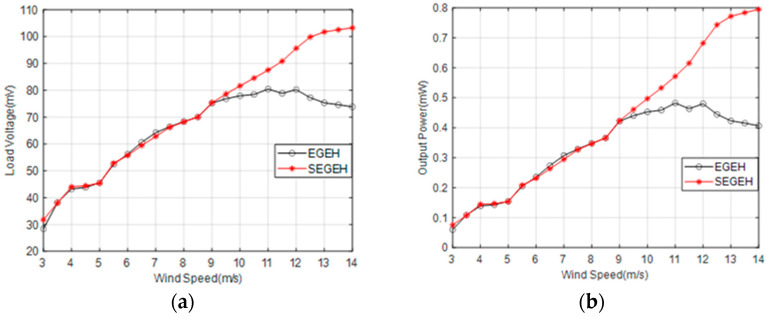
Load voltage and output power comparison of the SEGEH and the EGEH. (**a**) Load voltage. (**b**) Output power.

Figure 15 shows the variation of the SEGEH’s load voltage (a) and output power (b) when the load resistance is 13.2 Ω. At this point, the critical wind speed of the impact coupling between the bluff body and the spring was 7.5 m/s. Within the experiment’s wind speed range, the output voltage and output power of the SEGEH always increased as the wind speed increased. Compared with the EGEH, when the wind speed is less than 9 m/s, the output voltage and output power are basically the same. When the wind speed is greater than 9 m/s, the load voltage and output power of the SEGEH exceed that of the EGEH, and with the increase of wind speed, the load voltage and output power exceed more and more. When the wind speed is 14 m/s, the load voltage increased by 29.4 mV, thus demonstrating an increase of 39.8%. The output power increased by 0.38 mW, with an increase of 92.7%.

**Figure 16:**
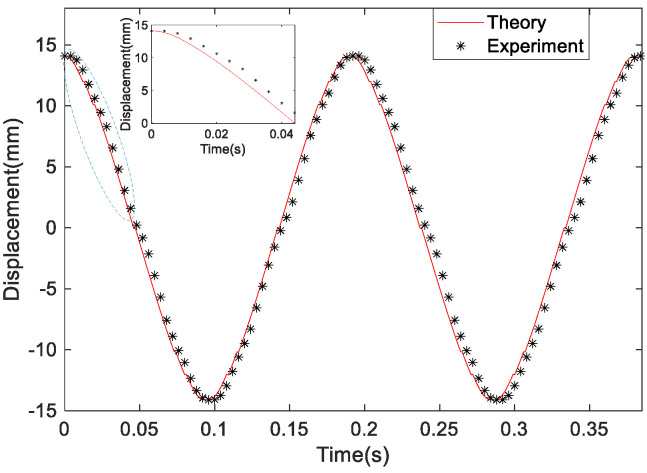
Comparison of the experimental displacement and theoretical displacement of the SEGEH at 10 m/s.

**Figure 17:**
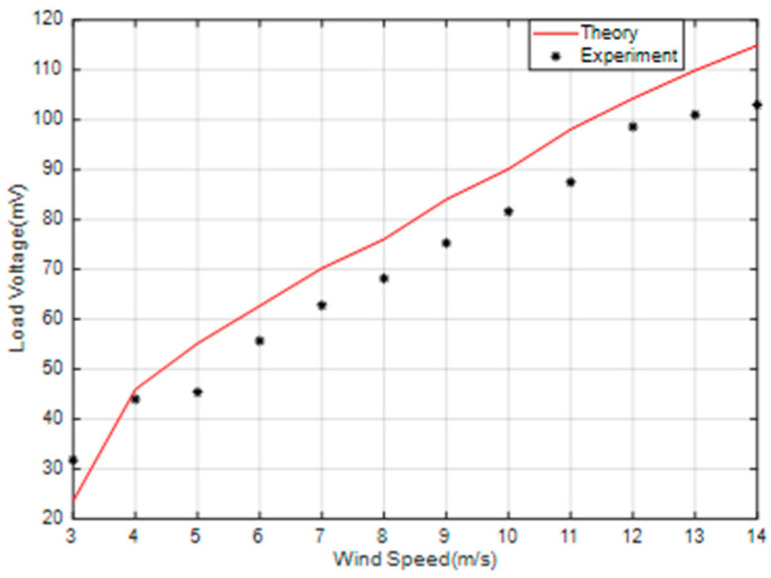
Comparison of the experimental voltage and theoretical voltage of the SEGEH.

Figure 16 shows the comparison of the experimental displacement and the theoretical displacement of the SEGEH at a wind speed of 10 m/s. The vibration of the bluff body is periodic, and the curve of every quarter period from the maximum amplitude to the center position is drawn by the three equations shown in Formulas (8) and (9). Taking the amplitude position as the initial position, the first curve is the second equation of Formula (8), with initial values of z(0) = 14.1 mm and v(0) = 0. z(0) is the experimental displacement measured by a high-speed camera. Then, when the displacement was 12 mm, t = 0.012 s, and the corresponding velocity, v(0.012) = −0.296 m/s, were obtained using numerical calculations, which were used as the initial values of the first equation in Formula (8) so as to obtain the second curve. Then, when the displacement was 10 mm, t = 0.018 s, and the corresponding velocity, v(0.018) = −0.336 m/s, were obtained using numerical calculations, which were used for the initial values of Equation (9); then, when the displacement was 0, it was obtained by numerical calculations. During the 1/4 vibration period, the obtained displacement curve is 1/4 of the displacement curve. The displacement curves with more periods can be obtained using the same method. Figure 17 shows the comparison between the experimental voltage and the theoretical voltage of the SEGEH at different wind speeds. The overall change trend is basically the same.

**Figure 18:**
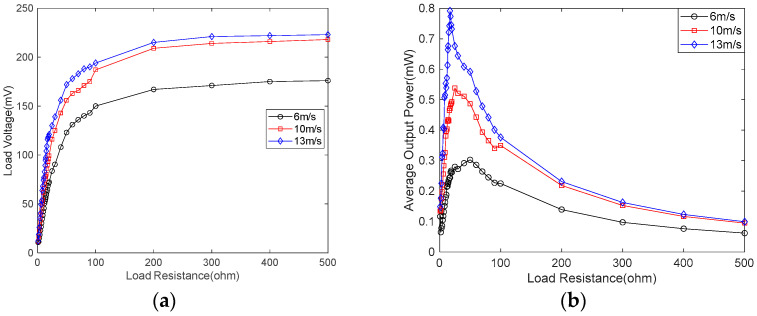
Load voltage and average output power of the SEGEH at different wind speeds and loads. (**a**) Load voltage. (**b**) Average output power.

Figure 18 shows the load voltage and the average output power of the SEGEH at different wind speeds and loads. The load voltage was measured by changing the load resistance at different wind speeds, so as to determine the optimal load resistance at the maximum output power. As shown in Figure 18a, the load voltage of the SEGEH increased rapidly at first and then it remained almost constant as the load resistance increased. The output power first increased and then decreased as the load resistance increased. The optimal load resistance was variable, and it tended to decrease with the increase in wind speed. As shown in Figure 18b, when the wind speed is 6 m/s, 10 m/s, and 13 m, respectively/s, the optimal load resistance of the SEGEH is 50 Ω, 25 Ω, and 17 Ω, respectively, which gradually decreased as the wind speed increased. Therefore, if the SEGEH is to achieve the best power output, it is necessary to determine the best load resistance by considering its working wind speed comprehensively, or by dynamically adjusting its load resistance in accordance with the wind speed.

## 4. Conclusions

In this paper, a spring-coupling electromagnetic galloping energy harvester is proposed. Based on the galloping characteristics of the large amplitude, the vibration energy without an induced electromotive force is converted into elastic energy using the coupling spring, and the output characteristics mean that the output voltage and output power always increase as the increase in wind speed is obtained.

Each vibration period of the SEGEH is divided into two phases: the independent galloping phase and the impact coupling phase. During the impact coupling phase, the coupling spring can convert the vibration energy consumed by the vibration stroke, without inducing an electromotive force output from the bluff body, into the elastic energy of the spring, which provides an elastic force for the return of the bluff body, it increases the vibration speed, and it improves the duty cycle of the induced electromotive force and the output power of the energy harvester. Regarding the laboratory wind tunnel platform, this study first selected nine springs with different parameters for coupling impact experiments, and the feasibility of the SEGEH design scheme was determined. In accordance with the experimental results, the 0.6 × 12 × 25 spring was selected as the coupling spring of this experiment, and then the output characteristics of the spring were studied. The results show that the stiffness of the coupling spring and the initial distance between the coupling spring and the bluff body will affect the output characteristics of the SEGEH. The SEGEH with the 0.6 × 12 × 25 spring can realize the output characteristic of the output voltage, in that it always increases as the wind speed increases, within the range of the experimental wind speed. When the wind speed is greater than 9 m/s, the output voltage and output power of the SEGEH exceed that of the EGEH, and the excess voltage and power continues to increase with the increase in wind speed. When the wind speed is 14 m/s, the output voltage is 103.2 mV, and the output power is 0.79 mW. The output voltage increased by 29.4 mV, and the output power increased by 0.39 mW, with an increase rate of 39.8% and 92.7%, respectively. This scheme is a new type of electromagnetic galloping energy harvester, and the method of reducing the amplitude and increasing the output duty cycle has general significance for improving the electromagnetic galloping energy harvester.

## Figures and Tables

**Table 1:** Material properties and structural parameters of the experimental prototype.

Structure	Parameters	Value
Cantilever beam	Young modulus (GPa)	110
	Poisson ratio	0.34
	Dimensions of the cantilever beam (mm)	48 × 12 × 0.2
Permanent magnet	Thickness (mm)	5
	Diameter(mm)	10
	Residual flux density (T)	0.36
Coil	Number of turns	300
	Length (mm)	10
	Inner diameter (mm)	10
	Wire diameter (mm)	0.2
	Resistance (Ω)	13.2
Stiffness coefficient of the coupling springs	0.6 × 12 × 25(mm)	123
	0.5 × 12 × 25(mm)	92
	0.4 × 12 × 25(mm)	17
Bluff body	h × d × d (mm)	35 × 15 × 15
The gap between the coil and the bluff body	(mm)	0.7
Load resistance	(Ω)	13.2

## Data Availability

Not applicable.
